# Chromatin-Associated Molecular Patterns (CAMPs) in sepsis

**DOI:** 10.1038/s41419-022-05155-3

**Published:** 2022-08-12

**Authors:** Colleen P. Nofi, Ping Wang, Monowar Aziz

**Affiliations:** 1 Center for Immunology and Inflammation, The Feinstein Institutes for Medical Research, Manhasset, NY USA; 2 Elmezi Graduate School of Molecular Medicine, Manhasset, NY USA; 3 Department of Surgery, Zucker School of Medicine at Hofstra/Northwell, Manhasset, NY USA; 4 Department of Molecular Medicine, Zucker School of Medicine at Hofstra/Northwell, Manhasset, NY USA

**Keywords:** Cell death and immune response, Apoptosis

## Abstract

Several molecular patterns have been identified that recognize pattern recognition receptors. Pathogen-associated molecular patterns (PAMPs) and damage-associated molecular patterns (DAMPs) are commonly used terminologies to classify molecules originating from pathogen and endogenous molecules, respectively, to heighten the immune response in sepsis. Herein, we focus on a subgroup of endogenous molecules that may be detected as foreign and similarly trigger immune signaling pathways. These chromatin-associated molecules, i.e., chromatin containing nuclear DNA and histones, extracellular RNA, mitochondrial DNA, telomeric repeat-containing RNA, DNA- or RNA-binding proteins, and extracellular traps, may be newly classified as chromatin-associated molecular patterns (CAMPs). Herein, we review the release of CAMPs from cells, their mechanism of action and downstream immune signaling pathways, and targeted therapeutic approaches to mitigate inflammation and tissue injury in inflammation and sepsis.

## Facts

- Extracellular nucleic acids (DNA and RNA) and their associated molecules present distinct molecular patterns that activate immune cells.
- Increased blood levels of CAMPs correlate with the severity of sepsis.
- Extracellular TERRA could be a novel CAMP.
- Targeting CAMPs with neutralizing antibodies, receptor inhibitors, and small molecule inhibitors attenuate inflammation in sepsis.

## Open questions

- Do extracellular histones and eCIRP exist in different post-translational forms?
- Do epigenetic changes influence the release and functions of CAMPs?
- Can glycoRNAs induce or regulate inflammation in sepsis?
- Are ribosomes released to act as CAMPs in sepsis?

## Introduction

Sepsis is a complex inflammatory disorder initiated by a dysregulated host immune response to infection [1]. In the United States, approximately 1.7 million adults are affected each year causing more than 250,000 deaths [2, 3]. The innate immune system is activated by pathogen-associated molecular patterns (PAMPs) and damage-associated molecular patterns (DAMPs), triggering inflammation [4, 5]. Intracellular proteins, nucleic acids, and lipids originating from the nucleus, cytoplasm, mitochondria, and granules fall into the broad category of DAMPs [4, 6, 7]. PAMPs can be further separated into MAMPs and NAMPs, containing the molecular patterns derived from microbes and nematodes, respectively [8, 9]. In addition, lifestyle-associated inflammatory diseases and their corresponding molecular patterns (LAMPs) have been distinguished from conventional DAMPs [10]. Self-associated molecular patterns (SAMPs) maintain cellular homeostasis and regulate innate immune cells when they become activated [11]. To simplify this broad area of DAMPs, the molecules derived from the nucleus or molecules associated with chromatin can be designated as chromatin-associated molecular patterns (CAMPs). Thus, by name, CAMPs refer to chromatin made up of nucleosomes containing DNA and histones. However, several nucleic acids and proteins, i.e., mitochondrial DNA, cell-free (cf) RNAs, microRNAs, telomeric repeat-containing RNA (TERRA), extracellular traps (ETs), and RNA- or DNA-binding proteins can also be included in this group, given their similar origins’ contribution to inflammation and organ dysfunction in sepsis. Increased levels of CAMPs, i.e., cfRNA, cfDNA, histones, extracellular cold-inducible RNA-binding protein (eCIRP), high mobility group box 1 (HMGB1), and ETs in blood have been shown to correlate with disease severity in sepsis (Table 1) [12–17].

**Table 1:** CAMPs in Experimental and Clinical Sepsis.

CAMPs	Sepsis	
	Experimental	Clinical
**cfDNA**	Plasma cfDNA is increased in *E. Coli*-injected and CLP-induced septic mice [159, 160].	Plasma cfDNA correlated with severity of septic patients and ICU mortality [50]. Serum levels of cfDNA are elevated and predict prognosis and ICU mortality in sepsis [13, 161]. cfDNA levels correlated with sepsis severity and organ dysfunction [12]. cfDNA marker of sepsis severity and prediction of inflammatory second hit in ICU patients [162].
**mtDNA**	mtDNA induces systemic and lung inflammation when administered intravenously in rats [68].	Elevated in the plasma of septic patients and correlated with disease severity [70].
**ETs**	Neutrophil, basophil, eosinophil, and macrophage ETs have been implicated in pathologic inflammation [73, 91, 163]. NETs are increased in alveolar spaces and microvasculature in murine LPS-induced endotoxemia [164].	Increased plasma levels of NETs correlated with organ dysfunction in septic patients [12].
**Histones**	H3 is released in LPS-induced endotoxemia, and increased plasma levels are associated with severity of shock [165].	Increased circulating levels in septic patients [15].
**exRNA**	exRNAs induce inflammatory responses in inflammatory models [116].	Levels of exRNA are elevated in the serum of septic patients [16].
**miRNA**	In the serum of CLP mice, exosomes showed elevated levels of miR-16, miR-17, miR-20a, miR-20b, miR-26a, and miR-26b [166].	Elevated circulating levels of miRNAs in patients with sepsis [167].
**eCIRP**	Mediator of injury and inflammation in preclinical models of sepsis and shock [20].	Serum levels are elevated in septic patients and predictive of sepsis severity and overall mortality [17].
**HMGB1**	Increased levels in LPS-induced endotoxemia [168]. Significantly increased in CLP-induced sepsis and associated as a late mediator of inflammation with inflammatory cytokines [150].	Serum levels are elevated in human patients with bacteremia and sepsis-induced organ dysfunction [14]. Elevated in pneumonia-induced sepsis and associated with mortality [169].
**TERRA**	cfTERRA stimulates inflammatory cytokines when incubated with immune-responsive cells [129].	Identified in human blood and tissue [170].

*cfDNA* Cell-free DNA, *mtDNA* Mitochondrial DNA, *ETs* Extracellular traps, *NETs* Neutrophil extracellular traps, *exRNA* Extracellular RNA, *miRNA* Micro-RNA, *eCIRP* Extracellular cold-inducible RNA-binding protein, *HMGB1* High mobility group box 1, *TERRA* Telomeric repeat-containing RNA.

**Fig. 1:**
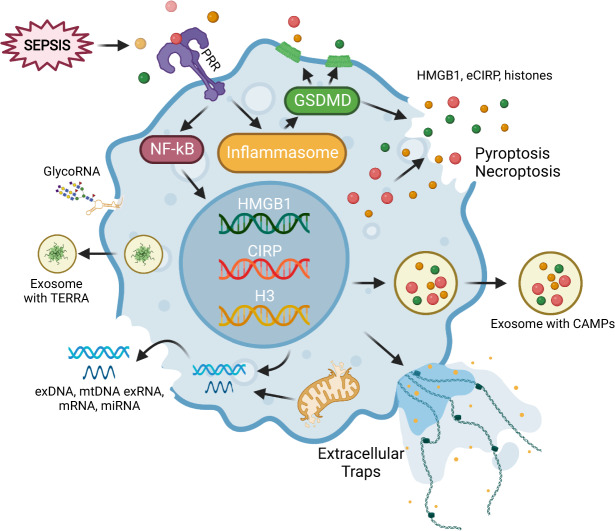
Release of CAMPs in sepsis. Septic insults, PAMPs, and other noxious stimuli activate immune cells (macrophages) to increase the expression and release of CAMPs, i.e., nuclear and mitochondrial DNAs, histones, RNAs, miRNAs, extracellular traps, HMGB1, and eCIRP. CAMPs are released through active processes like exosomes and GSDMD-mediated pores and passive release mechanisms like pyroptosis, necroptosis, ETosis, and secondary necrosis. PRR Pattern recognition receptor, GSDMD Gasdermin D, HMGB1 High mobility group box 1, eCIRP extracellular CIRP, CAMPs chromatin-associated molecular patterns, exDNA extracellular DNA, mtDNA Mitochondrial DNA, TERRA Telomeric repeat-containing RNA.

**Fig. 2:**
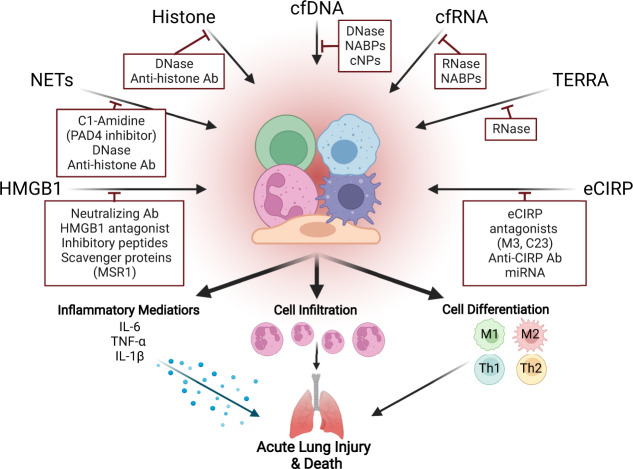
Targeting CAMPs to attenuate inflammation and acute lung injury (ALI) in sepsis. Several inhibitors, i.e., neutralizing antibodies targeting CAMPs or their receptors, small molecule inhibitors, like C23 and M3, CI-Amidine, endogenous inhibitor like miRNAs, RNases, DNase, scavenging molecules, i.e., cNP, NABPs, MSR1 have been discovered to counteract CAMPs, thereby inhibiting the release of inflammatory mediators, cellular infiltrations, and differentiation and inhibit the development of ALI in sepsis. cfDNA Cell-free DNA, TERRA Telomeric repeat-containing RNA, eCIRP Extracellular CIRP, cNP Cationic nanoparticles, NABPs Nucleic acid-binding polymers, MSR1 Macrophage scavenger receptor 1.

CAMPs are released from cells through active and passive release pathways. Among the active release processes, exosomal, lysosomal, and gasdermin D (GSDMD) pores (pre-pyroptotic) comprise the primary mechanisms for CAMP release while keeping cells alive [18–21]. By contrast, passive release mechanisms include secondary necrosis, necroptosis, pyroptosis, and NETosis [18, 22, 23]. In septic conditions, once released from cells, CAMPs recognize their receptors, i.e., Toll-like receptor 4 (TLR4), TLR2, TLR7, TLR9, receptor for advanced glycation end products (RAGE), triggering receptor expressed on myeloid cells-1 (TREM-1), CD24, and cytosolic DNA sensors absent in melanoma 2 (AIM2), and cyclic GMP-AMP synthase (cGAS)-stimulator of interferon genes (STING) to activate immune cells, causing inflammation and tissue injury. [6, 7, 24–26] Conversely, neutralizing antibodies or antagonists against those receptors have been shown to attenuate CAMP-induced inflammation [6, 7]. In this review we will discuss molecules related to CAMPs, how CAMPs are released from cells during inflammation, and the signal transduction pathways involving CAMP-mediated inflammation in experimental and clinical sepsis (Fig. 1; Table 2). Finally, we will emphasize targeting CAMPs to abrogate inflammation in pre-clinical conditions (Fig. 2; Table 3).

**Table 2:** CAMPs Signal Transduction Pathways.

CAMPs	Sensors	Subcellular Localization of Sensors	Signaling Pathways/ Transcription Factors	References
**cfDNA**	TLR9	Endosomes	NF-κB/AP1/CREB/IRF3/IRF7	[34, 171, 172]
	AIM2	Cytosol	ASC/Caspase-1	[34, 171]
	IFI16	Nucleus/cytosol	IRF3/IRF7	[34, 43]
	cGAS	Cytosol	NF-κB/IRF3/IRF7	[34, 173]
	STING	Endoplasmic reticulum	NF-κB/IRF3/IRF7	[34, 173, 174]
	RAGE	Plasma membrane	NF-κB/AP1/STAT3	[171]
**mtDNA**	TLR9	Endosomes	NF-κB/AP1/CREB/IRF3/IRF7	[171, 172]
	cGAS	Cytosol	NF-κB/IRF3/IRF7	[173]
	STING	Endoplasmic reticulum	NF-κB/IRF3/IRF7	[173, 174]
**NETs**	cGAS	Cytosol	NF-κB/IRF3/IRF7	[173]
**Histones**	TLR2	Plasma membrane	NF-κB/AP1/CREB	[34, 171, 172]
	TLR4	Plasma membrane	NF-κB/AP1/CREB/IRF3/IRF7	[34, 171, 172]
	TLR9	Endosomes	NF-κB/AP1/CREB/IRF3/IRF7	[34, 171, 172]
	NLRP3	Cytosol	ASC/Caspase-1	[34, 171]
**exRNA**	TLR3	Endosomes	NF-κB/ATF2/c-Jun/IRF3	[171, 172, 175]
	TLR7	Endosomes	NF-κB/ATFs/c-Jun/IRF7	[171, 172, 176]
	TLR8	Endosomes	NF-κB/ATFs/c-Jun/IRF7	[171, 172, 176]
	NLRP3	Cytosol	ASC/Caspase-1	[171]
	RAGE	Plasma membrane	NF-κB/AP1/STAT3	[171]
	RIG1	Cytosol	NF-κB/IRF7/IRF3	[177]
	MDA5	Cytosol	NF-κB/IRF7/IRF3	[177]
**TERRA**	TLR9	Endosomes	NF-κB/AP1/CREB/IRF3/IRF7	[171, 172]
**glycoRNAs**	Siglecs	Plasma membrane	SHP-1/SHP-2/NF-κB	[131]
**eCIRP**	TLR4	Plasma membrane	NF-κB/AP1/CREB/IRF3/IRF7	[171, 172]
	TREM-1	Plasma membrane	NF-κB/AP1/NFAT	[171]
	NLRP3	Cytosol	ASC/Caspase-1	[171]
**HMGB1**	TLR2	Plasma membrane	NF-κB/AP1/CREB	[34, 171, 172]
	TLR4	Plasma membrane	NF-κB/AP1/CREB/IRF3/IRF7	[34, 171, 172]
	TLR9	Endosomes	NF-κB/AP1/CREB/IRF3/IRF7	[34, 171, 172]
	RAGE	Plasma membrane	NF-κB/AP1/STAT3	[34, 171]
	TREM-1	Plasma membrane	NF-κB/AP1/NFAT	[171, 178]

*cfDNA* Cell-free DNA, TLR toll-like receptors, *NF-kB* Nuclear factor kappa B, *IRF* Interferon regulatory factor, *AIM2* Absent in melanoma 2, *IFI16* Interferon-inducible protein 16, *cGAS* Cyclic guanosine monophosphate-adenosine monophosphate synthase, *STING* Stimulator of interferon genes, *mtDNA* Mitochondrial DNA, *NETs* Neutrophil extracellular traps, *NLRP3* NLR family pyrin domain containing 3, *exRNA* Extracellular RNA, *RAGE* Receptor for advanced glycation end products, *RIG1* Retinoic acid inducible gene I, *MDA5* Melanoma differentiation-associated protein-5, *miRNA* micro-RNA, *eCIRP* Extracellular cold-inducible RNA-binding protein, *TREM-1* Triggering receptors expressed on myeloid cells-1, *HMGB1* High mobility group box 1, *TERRA* Telomeric repeat-containing RNA.

**Table 3:** Targeting CAMPs in Sepsis.

CAMPs Target	Therapeutics/ Antagonists	Sepsis Models in Mice	Impacts	References
**cfDNA**	DNase	*E. Coli*	Reduced inflammation, reduced weight loss, and improved survival	[179]
	cNP (PEI-g-ZIF)	CLP	Reduced systemic inflammation and organ injury, reduced weight loss, and improved clinical outcomes and survival	[180]
	NABPs, PANAM-G3	CLP	Ameliorated organ injury and improved survival	[58]
	NABN, MSN-PEI	CLP	Inhibited inflammation and reduced organ injury	[58]
	PAD4 inhibitor, C1 Amidine	CLP	Improved survival, increased serum IL-10 and decreased splenic IL-6	[181]
**exRNA**	RNase	CLP	Reduced cardiac apoptosis and septic cardiomyopathy, reduced renal dysfunction and hepatocellular injury, and reduced systemic inflammation	[16]
**NETs**	DNase	LPS, CLP	Reduced intravascular thrombin activity, reduced platelet aggregation, and improved microvascular perfusion Reduced NET formation, decreased systemic inflammation and decreased lung injury Reduced cfDNA and impeded early immune response	[12, 81, 182, 183]
	PAD4 inhibitor, C1 Amidine	CLP	Decreased NET formation and improved survival.	[181]
	PAD2/PAD4 inhibitor, YW3-56	LPS	Reduced citrullinated H3 production and NET formation in lungs, alleviated ALI and improved survival	[184]
**Histones**	Anti-histone Antibody	CLP	Improved survival	[185]
	Anti-histone Antibody, H4	LPS, CLP	Improved survival	[15]
	PAD4 inhibitor, C1 Amidine	CLP	Inhibited histone citrullination and improved survival	[181]
	APC	*E. coli	Improved survival	[15]
	Histone deacetylase inhibitor, SAHA	LPS	Decreased systemic inflammation and improved survival	[186]
	Histone deacetylase inhibitor, SAHA	CLP	Decreased cytokine storm, decreased acute liver injury and improved survival	[187]
**eCIRP**	C23, CIRP-derived peptide inhibitor	Cecal slurry	Reduced systemic and pulmonary inflammation in neonatal sepsis	[188]
	C23, CIRP-derived peptide inhibitor	CLP	Decreased systemic, lung, and kidney injury, decreased inflammation, and improved survival	[189]
	C23, CIRP-derived peptide inhibitor	Intestinal I/R	Attenuated systemic and lung inflammation and reduced intestinal tissue injury	[190]
	M3, CIRP derived peptide inhibitor	LPS, CLP	Decreased systemic inflammation, improved lung injury, and improved survival	[134]
	M3, CIRP derived peptide inhibitor	Intestinal I/R	Reduced local and systemic inflammation in the serum, lung, and intestines	[191]
	M3, CIRP derived peptide inhibitor	Cecal slurry	Cardioprotective, decreased systemic and pulmonary inflammation, and improved survival	[192]
	miR130b-3p mimic	CLP	Attenuated systemic inflammation and acute lung injury in sepsis	[125]
**HMGB1**	Anti-HMGB1 neutralizing antibodies	LPS, CLP	Improved cytokine profile, increased resistance to secondary bacterial infections, and improved survival Decreased systemic inflammation and improved lethality Improved neutrophil function and reduced post-sepsis immunosuppression	[14, 156, 193, 194]
	Anti-HMGB1 neutralizing antibodies	Surgical induction peritonitis	Improved survival and protected against organ injury	[150]
	DNA-binding A box	LPS, CLP	Protected against organ injury and improved survival	[150]
	RAGE antagonist, recombinant box A	CLP	Reduced systemic inflammation and improved survival	[195]
	HMGB1 antagonist, Ethyl pyruvate	LPS, CLP	Reduced systemic inflammation and improved survival	[196]
	HMGB1 antagonist, Nicotine	LPS, CLP	Attenuated clinical manifestations of endotoxemia and improved survival	[197]
	HMGB1 antagonist, Chloroquine	LPS, CLP	Inhibited cytokine activity and improved survival	[198]
	HMGB1 antagonist, Zingerone	CLP	Reduced tissue injury and improved survival	[199]
	HMGB1 antagonist, Glycyrrhizin	LPS	Protected against endotoxemia and liver damage	[200]
	HMGB1 antagonist, Haptoglobin	CLP	Improved systemic inflammation and clinical signs, and improved survival	[201]
	HMGB1 antagonist, stearoyl LPC	LPS, CLP	Attenuated inflammation and improved survival	[202]
	Inhibitory peptide, HPep1	LPS	Reduced systemic inflammation	[156]
	Scavenger receptor competitive inhibitor, M-BSA	LPS	Reduced inflammation and improved survival	[157]
	siRNA against HMGB1	CLP	Alleviated cytokine storm and improved survival	[203]

^*^In baboons. *cfDNA* Cell-free DNA, *CLP* Cecal ligation and puncture, *cNP* cationic nanoparticles, *NABPs* Nucleic acid-binding nanoparticles, *PANAM-G3* Polyamidoamine dendrimer, *NABN* Nucleic acid-biding nanoparticle, *MSN-PEI* Mesoporous silica nanoparticle functionalized with polyethylenimine, *exRNA* extracellular RNA, *NETs* Neutrophil extracellular traps, *LPS* Lipopolysaccharide, *SAHA* Suberoylanilide hydroxamic acid, *APC* Activated protein C, *eCIRP* Extracellular cold-inducible RNA-binding protein, *miRNAs* Micro-RNAs, *HMGB1* High m obility group box 1, *RAGE* Receptor for advanced glycation end products, *LPC* Lysophosphatidyl-choline, *M-BSA* Maleylated bovine serum albumin.

## Chromatin-associated molecular patterns (CAMPs)

Chromatin is a structural complex of DNA with various basic and acidic proteins. Histones are basic, positively charged proteins that associate with negatively charged DNA, thereby organizing DNA into structures called nucleosomes [27, 28]. A nucleosome consists of a 147 bp DNA sequence wrapped around a set of eight histones called an octamer. Each histone octamer consists of two copies of each of the histone proteins, H2A, H2B, H3, and H4. The linker histones, H1, sit on the ends of DNA, keeping DNA correctly wrapped with core histones [27, 28]. Chromatin’s primary function is to compress DNA into a compact unit that fits within the cell’s nucleus. Chromatin increases genome stability and hinders the enzymes needed for gene transcription [27–29]. The phenomena of chemical modifications of histones and DNA, called epigenetic marks, change chromatin structure and expose regulatory elements for transcription factors to bind and impact gene expression [29, 30]. Chromatin modifications link with various cell processes, including DNA replication, transcription, DNA repair, genetic recombination, and cell division [29–31]. Following stress, infection, injury, or other inflammatory stimuli, chromatin (and associated states of nucleic acids) are released from cells, acting as CAMPs to augment inflammation and tissue injury. CAMPs include but are not limited to extracellular DNA, histones, RNA, microRNA (miRNA), mitochondrial DNA, eCIRP, HMGB1, and ETs.

## Extracellular DNA

During cell death, such as apoptosis, necrosis, or ETosis, DNA exits the cell. Extracellular or cell-free DNAs (cfDNAs), including pathogen-derived CpG, damaged cell-released nuclear or mitochondrial DNA, and ETs, have been reported to not only represent biomarkers of sepsis but also contribute to the length and severity of the inflammatory response in immune cells [32]. While bacterial DNA can activate the immune system through its CpG motifs, mammalian DNA is ordinarily inactive and only acquires activity once released extracellularly [32]. DNA can be associated with nuclear, cytoplasmic and serum proteins, which can promote its uptake intracellularly to stimulate internal DNA sensors [33].

The effect of DNA to serve as a CAMP necessitates the transfer of DNA from one cell to the extracellular space and then uptake into another cell [34]. There are various mechanisms by which DNA may then be recognized and trigger downstream signaling pathways that lead to inflammation. The three major receptors responsible for DNA-driven immune responses include toll-like receptor 9 (TLR9), absent in melanoma 2 (AIM2) and cyclic-GMP-AMP synthase (cGAS). TLR9 is expressed on the endosomal membrane. It functions as a DNA receptor that specifically recognizes hypomethylated CpG motifs and induces type-I interferon (IFN) as well as other inflammatory genes [35]. The coupling mechanisms of cytosolic DNA to its downstream pro-inflammatory signaling cascades is multifold. Cytosolic DNA can drive the Nlrp3 inflammasome or PYHIN inflammasomes assembly and activation, including AIM2, and interferon-inducible protein 16 (IFI16). Upon inflammasome activation, DNA sensors recruit adaptors to activate caspase-1, leading to proteolytic cleavage and release of active forms of IL-1β and IL-18 [36, 37]. Perhaps most significant in cytosolic DNA sensing is the DNA-binding protein, cGAS. cGAS is responsible for recognizing self- verse other sources of chromatin (i.e., CAMPs) and initiating a robust response to their accumulation in the cytosol through activation of STING [38, 39]. Recent work has highlighted the integral role of cGAS in distinguishing sources of DNA. For example, the binding of nuclear chromatin to histones H2A and H2B, and through selective suppression of cytosolic cGAS through the cell cycle have been shown to prevent aberrant activation of cGAS [38, 40]. For non-self-DNA in the cGAS-STING pathway, DNA binding activates cytosolic cGAS to generate the second messenger cyclic GMP-AMP (cGAMP), which binds to the endoplasmic reticulum-localized adaptor protein STING. After activation, STING translocates to the Golgi and recruits kinases such as TANK-binding kinase 1 (TBK1) and IkB kinase (IKK) which phosphorylate interferon regulatory factor 3 (IRF3) and the NFkB inhibitor IkBα. TBK1 acts as a convergence point for multiple PRR-driven pathways in IRF3 phosphorylation and eventual transcriptional activation of type-I IFN and related genes. Notably, besides acting as an adaptor for DNA sensing, STING is also capable of acting as a direct sensor for secondary messenger molecules including cyclic di-AMP (c-di-AMP) and cyclic di-GMP (c-di-GMP), thereby serving as a strong inducer of type-I IFN [33]. Other known DNA-binding proteins may similarly mediate DNA-induced type-I IFN and pro-inflammatory cytokine production. These DNA-binding proteins include DNA-dependent activator or IRFs (DAI) [41], RNA polymerase III [42], IFI16 [43], oligodeoxynucleotides (including DHX36 and DHX9) [44, 45], and DDX41 [46]. Finally, additional DNA binding proteins may be involved in cytosolic DNA sensing leading to production of type 3 interferon and IFNβ, including Ku70 and leucine-rich repeat in flightless-I interacting protein (LRRFIP1), respectively [47–49].

In human sepsis, plasma levels of cfDNA are elevated, and high plasma DNA is linked to increased mortality in sepsis [50]. Endogenous DNases comprise the first line of defense against DNA functioning as a CAMP. Studies of mice lacking these enzymes demonstrated overexpression of type-I IFNs in macrophages and led to embryonic lethality in mice in STING-dependent manner, suggesting potential therapeutic strategies targeted at DNA as a CAMP in sepsis [51]. Further, experimental models targeting components of these signaling pathways has been shown to have beneficial effect. For example, STING knockout mice were protected in CLP-induced sepsis through reduced coagulation [52]. Similarly, models utilizing mice deficient in AIM2 [53], TLR9 [54], TLR4 [55], IRF3 [56], and related proteins were protected in experimental sepsis models, suggesting further therapeutic potential. Other strategies have investigated the use of ~40 nm cationic nanoparticles (cNP) to scavenge cfDNA and inhibit the activation of primary monocytes [57]. In the context of sepsis, nucleic acid-binding nanoparticles (NABPs) aiding cfDNA clearance exhibited beneficial outcomes [58]. This suggests a different direction of nanomedicine in treating inflammatory pathologies, including sepsis [57, 58].

## Mitochondrial DNA

Mitochondrial DNA (mtDNA) is another source of extracellular DNA that serves as a CAMP [59]. mtDNA released from the mitochondrial compartment to the cytoplasm sense intracellular DNA sensors to induce inflammation [60, 61]. Human mitochondria have evolved from endosymbiotic bacteria and thus may express molecules that resemble bacterial products [62]. Because of this resemblance, extracellular mtDNA whose CpG motifs are also unmethylated can serve as mediators of inflammation. As a result of cell death or cell activation, whole mitochondria (including proteins and DNA) can be released extracellularly in a process termed extrusion and thereafter elicit an immune response [63, 64]. mtDNA repair mechanisms are essential to cope with mtDNA damage. mtDNA degradation may contribute to the exaggerated innate immune response by fragmented mtDNA. Several components of the mtDNA replication machinery, such as DNA polymerase γ, helicase Twinkle, and exonuclease (EXOG, ENDOG, or MGME1), as well as a major DNA-packaging protein mitochondrial transcription factor A (TFAM) play critical roles in maintaining mtDNA integrity [65].

Recent studies have further characterized the inflammatory cascade caused by mitochondrial extrusion, as it has been shown that mitochondria may act as a CAMP when released from cells undergoing necroptosis induced by TNFα [66]. For example, outside the cell, mtDNA causes mouse splenocytes to produce TNFα and bone marrow-derived macrophages to secrete IL-1β [59, 67]. mtDNA can stimulate various PRRs, including TLR9 [59, 67]. This endosomal receptor activates NF-κB- and IRF-mediated pro-inflammatory responses upon recognizing mtDNA and has been shown in animal models to induce inflammatory cytokine secretion by macrophages and neutrophil chemotaxis [68]. In addition to activation through TLR9, degraded intracellular mtDNA can also engage the cytosolic sensor, cGAS, and initiate STING signaling to trigger IFN responses [60, 69]. Furthermore, DNA has been shown to extend neutrophils lifespan. When stimulated with mtDNA, neutrophils have increased viability, which may contribute to excessive accumulation in tissues and initiate uncontrolled inflammation, causing poor outcomes in sepsis [70, 71]. Engulfment of released mitochondria has also been shown to alter macrophage production of cytokines and lead to dendritic cell maturation [34].

## Extracellular traps

Extracellular traps (ETs) refer to the process of chromatin reduction, breakdown of the nuclear envelope, and subsequent disruption of the extracellular membrane due to reactive oxygen species, resulting in the release of DNA structures [72, 73]. The most widely studied form of ET release is by neutrophils, the contents of which herein are referred to as neutrophil extracellular traps (NETs). One pathway involving NET formation by PAMPs and DAMPs is the activation of peptidyl arginine deaminase 4 (PAD4) via the TLR4 receptor [6, 74]. However, aside from TLR4/PAD4 pathway, NET formation is also mediated by Rho activation, cell cycle protein cyclin-dependent kinases 4 and 6 (CDK4/6) expression, and gasdermin D (GSDMD) pores [21, 75, 76]. Once released, various proteins can adhere to NETs, including CAMPs, such as histones, HMGB1, CIRP, myeloperoxidase (MPO), LL37, and over 30 components of primary and secondary granules, among which confer bactericidal activity [77–79].

ET’s role in maintaining host homeostasis is twofold. On the one hand, they can protect hosts from infectious diseases; however, on the other hand, they may cause pathologic alterations to induce tissue injury [6, 74]. During sepsis for example, neutrophil-endothelial cell interaction is crucial for promoting neutrophil infiltration into tissues; however, this interaction leads to increased NET formation [6, 74, 80]. Co-culture of neutrophils with endothelial cells has been shown to cause endothelial cell damage, which is attributed in part to excessive NETs, as NADPH oxidase inhibitors or DNase ameliorate endothelial dysfunction and cell damage [80]. NETs have been shown to play a crucial role in the pathogenesis of various pro-inflammatory conditions, including the promotion of intravascular thrombosis in disseminated intravascular coagulation, which has been shown to increase the morbidity and mortality in sepsis [6, 74, 81]. In various murine models of acute lung injury (ALI), increased levels of NETs, as well as histones H3 and H4, were found in the bronchoalveolar fluid [6, 74]. Administration of extracellular histones contained in NETs has been shown to increase damage to alveolar epithelial cells and the magnitude of ALI [6, 82]. Recent discovery has also shown NET-associated RNA to be a physiologically significant component of NETs. Specifically, RNA-LL37 sensing, subsequent cytokine release and self-propagating NET formation was shown to contribute to disease exacerbation in the inflammatory condition of psoriasis [83]. Along with NETosis, enzymes are released and have detrimental effects in promoting inflammation. Neutrophil elastase, a key component of chromatin degranulation, has been shown to increase the permeability of alveolar epithelial cells, whereas inhibiting this enzyme is beneficial in animal models of inflammation and associated ALI [84]. Serine proteases have also been shown to break down surfactants which are involved in the clearance of inflammatory cells and residual inflammation after ALI [82]. Finally, NETs can bind to the cytosolic DNA sensor, cGAS. Macrophages phagocytose NETs, and subsequently intracellular NETs’ DNA activates cGAS and induces type-I IFN production as previously described [85].

Akin to neutrophils, although comparatively fewer reports are available, recent studies have revealed ET formation by other immune cells including eosinophils, basophils, and macrophages upon induction with various stimuli including PAMPs, DAMPs, and cytokines. These forms of ETosis similarly involve the pathways used for NETosis [73, 86, 87]. Granulocytes including eosinophils and basophils exhibit similar release mechanisms and contents as NETs. Extracellular DNA released from eosinophils, or eosinophil extracellular traps (EETs) have been shown to be triggered by clinically relevant allergens and amplify inflammation. Clinically, EETs have demonstrated to be elevated in bronchoalveolar lavage fluid and associated with asthma severity in patients and are likely involved in triggering of other immune responses [73, 87]. Basophil extracellular traps (BETs) contain mitochondrial DNA, granule proteins and proteases and are released upon cytokine priming and complement activation [88]. Noting that BETs have been found in both human and mouse inflamed tissues, it is conceivable that they also play a role in inflammatory conditions [88].

Finally, macrophage extracellular traps (METs), have also been shown to entrap and kill microbes [89–91]. METs contain nuclear and mitochondrial DNA, MPO, and lysozyme proteins similar to NETs, EETs, and BETs [91–93]. Although MET release has been demonstrated in murine primary macrophages and macrophage cell lines, the release and impact of METs in human conditions is largely underexplored, however similar to NETs, METs likely play a role in disease pathologies [91].

Strategies to address excessive ET formation have demonstrated therapeutic potential in acute inflammatory conditions. For one, inhibition of NETosis via PAD4 deficiency or inhibition reduces the release of extracellular DNA, resulting in improved outcomes in sepsis [94]. PAD4 inhibitors, similar to chloroquine and APC, are early inhibitors targeting NET formation. On the other hand, late inhibitors of NETs, such as DNase or anti-histone antibodies have also been explored and demonstrated therapeutic potential. [6, 74, 94–96]

## Extracellular histones

Histones are cationic, intra-nuclear proteins that maintain the normal structure of chromatin [29–31]. In acute sterile organ injury, various toxic stimuli, including ischemic, traumatic, and hemorrhagic pathologies can result in cell death. Through this process and similar to DNA, histones and DNA-bound histones (nucleosomes) are released into the extracellular space by necrosis, apoptosis, and ETosis. Extracellular, DNA-free histones may be associated with histone chaperones or histone-associated factors to maintain their DNA-free form. Once in the extracellular space, histones (and related components) act as CAMPs, thereby promoting inflammation. Although Xu et al. demonstrated that intravenous injection of histones was lethal in mice [15], incredibly, little distinction has been made between the different forms of extracellular histones. This is in large part due to the limitations of available techniques to differentiate between free versus DNA-bound forms of histones. Importantly, the cytotoxicity and proinflammatory signaling induced by free histones compared to nucleosome-associated histones differ [97, 98].

Inflammatory phenomena of extracellular histones have been explored in various experimental conditions. For one, the toxic effect of histones has been demonstrated when added to endothelial cells [82, 97]. In experimental models of murine sepsis, in vivo studies have shown intravenously injected histones were lethal, whereas anti-histone antibodies reduced mortality [15]. Furthermore, sublethal doses of intravenously administered histones were pro-inflammatory, resulting in high levels of TNFα, IL-6, and IL-10 in a TLR4-dependent manner [15]. In human sepsis, levels of histones are significantly increased [99], and consistent with experimental murine models, appear to cause cellular injury in a TLR4-dependent manner [15, 100].

## Extracellular RNA

Extracellular RNAs (exRNAs) are a heterogeneous group of ribonucleic acids, including messenger (m), ribosomal (r), micro (mi), long non-coding (lnc), and circular (circ) RNAs. These RNAs can be released from cells into the extracellular space in free form, bound to proteins or phospholipids, or in association with extracellular vesicles (EVs) [101–103]. Analyses of EV-associated exRNAs revealed that miRNAs together with rRNAs comprise the most prevalent form of exRNA; however, by weight, rRNAs are the most abundant type in human plasma [104–107]. exRNAs associate with several proteins or ribonucleoprotein complexes and bind to high-density lipoproteins that protect them from degradation by extracellular RNases [108, 109]. More importantly, however, these protein interactions can impact the immunogenicity of exRNAs [109, 110]. Although in physiologic conditions only low levels of circulating exRNA can be detected in extracellular fluids, in acute states of cellular stress (such as in hypoxia, infection, and inflammation), the concentration of exRNAs is dramatically increased [107, 109, 111].

While different forms of exRNAs, including miRNAs and lncRNAs have been implicated in influencing inflammatory processes at different levels, recent work has revealed the influence of ribosomal exRNA as an important DAMP on cellular processes for leukocyte recruitment [101, 112]. For example, ribosomal exRNA may induce vascular hyper-permeability and vasogenic edema. This may be accomplished through activation of the vascular endothelial growth factor (VEGF) receptor-2 system, as well as through recruitment of leukocytes to the inflamed endothelium through M1-type polarization of inflammatory macrophages, or through exRNA serving as a pro-thrombotic cofactor thereby promoting thrombosis [101, 113]. In addition to sterile inflammation, exRNAs also augment the linking of bacteria to host cells, facilitating microbial invasion [114]. exRNAs function as CAMPs in a typical manner through recognition by membrane-bound PRRs including TLRs and RAGE as well as cytosolic receptors including retinoic acid-inducible gene-I (RIG-I), and melanoma differentiation-associated protein-5 (MDA-5) [115, 116]. The binding of exRNAs by such PRRs leads to the induction of different signaling pathways, resulting in the activation of transcription factors like c-Jun-N-terminal kinase or NF-κB and release of pro-inflammatory cytokines [117, 118]. Interestingly, ribosomal exRNAs fulfill several additional extracellular functions independent of recognition by PRRs, including the progression of cardiovascular diseases [101].

miRNAs are small endogenous non-coding RNAs that play a critical role in post-transcriptional regulation of gene expression by binding to complementary target mRNAs [119, 120]. miRNAs have been reported to affect immune processes, for example by inhibiting of NF-κB expression and modulating immune cell proliferation and differentiation [121]. Although the focus of miRNAs has revolved around their intracellular role, miRNAs have been readily found in the blood, with changes associated with acute physiologic stressors [105, 122, 123]. Many circulating miRNAs are bound to protective proteins (high-density lipoprotein and argonuate protein) or packaged into protective microvesicles (exosomes), as unbound miRNAs are rapidly degraded in the bloodstream [105, 108]. Compared to non-exosome associated miRNA, circulating miRNA found in exosomes are more likely modulated by stressor exposure. Stress-induced exosomal miRNA reductions have been correlated with increases in inflammatory proteins, thereby suggesting stress-modulated exosomes may be immune-stimulatory [105]. In a murine sepsis model, several miRNAs were found to be released into the blood via EVs. Compared to sham EVs, in septic EVs several miRNAs exhibited >1.5-fold increase. Specifically, these miRNAs included miR-126-3p, miR-122-5p, miR-146a-5p, miR-145-5p, miR-26a-5p, miR-150-5p, miR-222-3p, and miR-181a-5p. Furthermore, septic EVs were proinflammatory and increased IL-6, TNFα, IL-1β, and MIP-2 production via TLR7- and MyD88-dependent pathways [122].

In human sepsis, it has been demonstrated that miR-182, miR-143, miR-145, miR-146a, miR-150, and miR-155 were dysregulated in septic patients, and downregulation of specific miRNAs correlated with increased inflammatory cytokine production and monocyte proliferation [123, 124]. However, recently Guerin et al. revealed an unconventional function of extracellular miRNA to neutralize the action of a CAMP, eCIRP, because CIRP has a housekeeping role in interacting with RNAs [125]. Extracellular miRNA 130b‐3p mimic inhibited eCIRP‐induced inflammation in experimental models of sepsis. Although the damaging effect of eRNA can be counteracted by endogenous circulating RNase1, under acute inflammatory states, only the administration of exogenous, non-toxic RNase1 provides an effective and safe therapeutic regimen. Thus, novel in vitro and in vivo strategies, including natural endonucleases or synthetic nucleic acid binding/neutralizing polymers as antagonists, have been explored and show promise in combatting the destructive nature of eRNA [126, 127].

## TERRA, glycoRNAs, and extracellular ribosomes

Telomeres are the repetitive nucleotide regions found on chromosomal ends that protect DNA from decay. Telomeric repeat-containing RNA (TERRA), a lncRNA transcribed from telomeres, has been identified as a telomere-associated regulator of chromosome end protection [128]. Thus, intracellularly, TERRA plays a crucial role in telomere length homeostasis. A recent study reported that TERRA can be found in extracellular fractions in mouse tumor and embryonic brain tissue, as well as in human cell cultures that may stimulate the innate immune response [129]. Cell-free TERRA (cfTERRA) is a shorter form (∼200 nucleotides) of cellular TERRA and copurifies with CD63- and CD83-positive exosome vesicles. cfTERRA can also be found as a complex with histone proteins. Incubation of cfTERRA containing exosomes with peripheral blood mononuclear cells stimulated the expression of TNFα, IL-6, and C-X-C chemokine 10 (CXCL10) [129]. Although these findings with extracellular TERRA implicate a novel extrinsic function in tumor microenvironments, elucidation of extracellular TERRA in sepsis will direct a novel pathophysiology of inflammatory diseases.

Recently, highly conserved small lncRNAs, named glycoRNAs, were discovered [130]. These RNAs bear N-glycans in their structural backbone that are highly sialylated and fucosylated. GlycoRNAs are present in multiple cell types on the cell surface. They can interact with anti-dsRNA antibodies and members of the sialic-acid-binding immunoglobulin-like lectins (Siglecs) receptor family. Siglecs are expressed in various immune cells that recognize the sialic acid-containing ligands and initiate downstream signaling by activating Shp1 to negatively regulate TLR4 and B cell receptor (BCR) signaling pathways [131]. Since glycoRNAs are exposed to the external environment of cells, their interaction with various proteins could be possible, pinpointing their novel role in the immune system. A recent study identified the presence of small ribosomal subunit 40 S by negative stain transmission electron microscopy and velocity sedimentation in sucrose gradients of concentrated extracellular fractions [112]. Improved understanding of extracellular ribosomes could possibly implicate them as damage-associated molecular patterns or subclassify them as CAMPs.

## eCIRP

The RNA chaperone protein, CIRP, plays a critical role in upregulating the inflammatory cascade when released from cells as eCIRP in acute inflammatory conditions, including sepsis. Elevated plasma levels of eCIRP have been independently correlated with worse prognosis in human sepsis [20, 125]. Intracellular CIRP can be released outside the cell through various pathways. For one, CIRP can be released passively during necrotic cell death. In addition, in times of cellular stress, CIRP can be translocated from the nucleus to cytoplasmic stress granules and released extracellularly through exosome-mediated pathways, inflammasome-mediated GSDMD activation, pyroptosis, or necroptosis [18, 20]. Once released extracellularly, eCIRP recognizes its cognate receptor TLR4/MD2 complex expressed in several cell types, activating downstream NF-κB pathways and stimulating the release of pro-inflammatory cytokines [20, 132]. eCIRP has also been shown to stimulate the Nlrp3 inflammasome, leading to caspase-1 activation and subsequent expression of IL-1β and IL-18 and pyroptosis [6, 7, 132]. In addition to increasing pro-inflammatory cytokines, eCIRP has been shown to contribute to end-organ injury in sepsis and other acute inflammatory conditions [132].

In many cell types, including macrophages, lymphocytes, and neutrophils, eCIRP has been demonstrated to act as a CAMP in the context of cellular activation, cytokine and chemokine production, and NET formation. For example, injection of recombinant mouse (rm) CIRP leads to ALI in mice via macrophage, neutrophil, and endothelial cell activation and cytokine production in the lungs [133]. Furthermore, beneficial outcomes have been demonstrated through CIRP inhibition by using newly identified antagonists, C23 and M3, targeting its binding to TLR4 and TREM-1, respectively, or in CIRP knockout mice in various murine models of acute inflammatory conditions [6, 7, 132, 134]. In sepsis, therapeutic potential has been demonstrated by using anti-CIRP antibodies or CIRP-derived inhibitory peptides (C23 and M3) to prolong survival and attenuate end-organ injury [6, 7, 132, 134].

## HMGB1

The nuclear nonhistone chromatin-binding protein, HMGB1, plays a critical role in many intracellular functions, including the DNA replication and repair, regulation of transcriptional activity, and nucleosome formation [135]. When mobilized from the nucleus to the cytoplasm and then released extracellularly, HMGB1 becomes pro-inflammatory [14, 136]. The extracellular release of HMGB1 can occur actively through cytoplasmic vesicles or passively from necrotic cells (either alone or in complex with RNA, DNA, or nucleosomes) or through pyroptosis [14, 23, 136, 137]. Double-stranded RNA-dependent protein kinase (PKR) induces inflammasome activation and subsequent release of HMGB1 [137].

Extracellular HMGB1 activates innate immune cells to propagate pro-inflammatory signaling cascades. This occurs through recruitment of neutrophils to the site of tissue injury and through HMGB1 binding of other PAMPs, (including DNA, LPS, and lipoteichoic acid), which serves to potentiate their inflammatory impact [6, 7, 136, 138]. Furthermore, HMGB1 has been shown to bind to numerous cell surface receptors, including RAGE, TLR2, TLR4, TLR9, and TREM-1 [6, 7]. Binding of HMGB1 to these receptors leads to the activation of macrophages and endothelial cells and downstream production of pro-inflammatory chemokines, cytokines, and endothelial adhesion molecules [6, 7]. HMGB1 is markedly elevated in human sepsis and is widely known as a late mediator of sepsis, leading to greater morbidity, and mortality [135, 139]. HMGB1 has been shown to significantly attenuate erythropoietin (EPO)-mediated phosphorylation of the JAK2/STAT5 and mTOR signaling pathways, contributing to the chronic phase of anemia of inflammation [140]. As released extracellular HMGB1 can induce considerable inflammation and has demonstrated to cause detrimental effects globally in various disease states [141], many therapeutic strategies have been employed, supporting that targeting HMBG1 can improve outcomes in sepsis (including neutralizing antibodies, HMGB1 antagonists, and small inhibitory peptides) [6, 7].

## Detection of CAMPs in sepsis

**Fig. 3:**
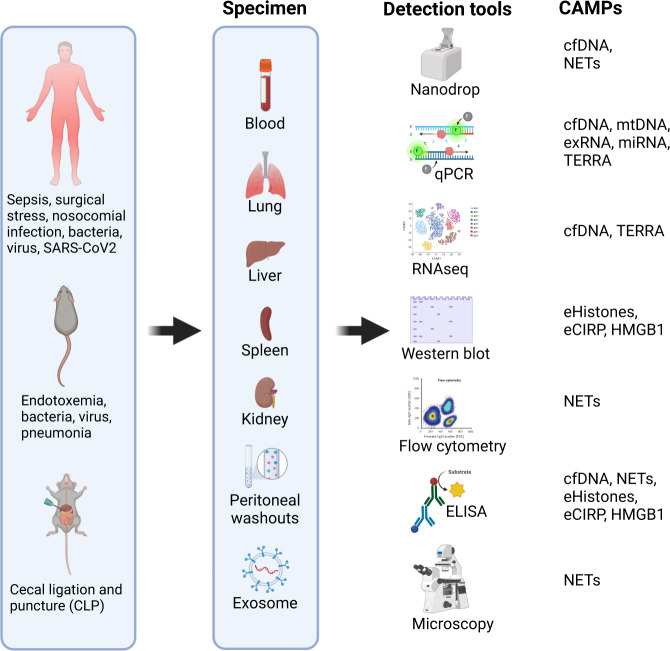
Detection of CAMPs in sepsis. For diagnostic and prognostic purposes, various samples of human and murine sepsis can be used to detect CAMPs using several immunological and molecular biological assay tools. CLP Cecal ligation, and puncture, NETs Neutrophil extracellular traps, eCIRP Extracellular CIRP, CAMPs, chromatin-associated molecular patterns; exDNA Extracellular DNA, mtDNA Mitochondrial DNA, TERRA Telomeric repeat-containing RNA.

Since CAMPs play a critical role in the immune response to infection, and elevated levels of CAMPs serve as diagnostic and prognostic markers in sepsis, assessment of CAMPs in biological fluids, i.e., the blood of experimental and clinical sepsis samples, is vital in determining the extent of the inflammation and tissue injury, monitoring disease progression, and elucidating potential treatment effects (Fig. 3). The levels of cfDNA in the plasma of sepsis patients are determined by real-time quantitative PCR (qPCR), quantitative PicoGreen fluorescence assay, and anti-dsDNA ELISA [142, 143]. Moreover, next-generation sequencing technology may be used to identify pathogens from cell-free plasma DNA of septic patients to overcome the shortcomings of traditional bacterial culture [144]. The presence of extracellular nuclear DNA (nDNA) and mtDNA in septic patients’ plasma are determined by qPCR using specific primers for nDNA and mtDNA [145]. Fragmented mtDNA in cells can be determined by staining cells with MitoTracker Red (mitochondria), TUNEL (fragmented DNA), and Hoechst (nucleus) [60]. Acknowledging that NETs contain DNA, citH3, and MPO, the plasma contents of NETs in sepsis and COVID-19 patients can be determined by PicoGreen fluorescence assay and ELISA by detecting citH3 and MPO using dsDNA Abs [146]. Immunohistochemistry can also be used to reveal NETs in lung samples, as was demonstrated in autopsy lung samples of COVID-19 patients to be exaggerated [147]. Circulating histones in septic patients’ sera are determined by ELISA [148]. Although it is tricky to distinguish the free verse nucleosomal histones, subtracting the values of DNA containing histones (determined by using dsDNA ELISA assays) from the values of total histones may indirectly provide amounts of free histones in the blood of septic patients. Extracellular RNA, especially the miRNA as free form or in EV contained form, are mainly detected by qPCR and microarray after isolating the total RNA from plasma samples [125, 149]. TERRA can be determined by RNA in situ hybridization assay [129]. Moreover, RNA-seq analyses revealed TERRA to be among the most highly represented transcripts in extracellular fractions extracted from normal and cancer patient blood plasma [129]. It has also been reported that cfTERRA can be identified by cyro-electron microscopy and ChIP assays [129]. The plasma levels of the DNA- and RNA-binding proteins HMGB1 and eCIRP in septic patients are measured by ELISA [20, 125, 150].

## Scavenging CAMPs to regulate inflammation

CAMPs may be released passively from necrotic cells. Since inefficient clearance of apoptotic cells may lead to necrosis and augmented CAMP release, molecules that contribute to apoptotic cells’ phagocytic clearance may help reduce circulating CAMPs. Milk fat globule-EGF factor VIII (MFG-E8) plays a critical role in efferocytosis, and thus may be implicated in the regulation of CAMPs [151]. Intracellular DNase and nucleic acid-binding polymers (NABPs) have also been shown to play a pivotal role in the degradation of DNA in apoptotic cells or engulfed cells [58, 152]. Furthermore, other scavenging molecules, as have been described in their role for scavenging dead cells, debris, and DAMPs from the extracellular compartment, may also be implicated in the regulation and removal of CAMPs as means for ameliorating inflammation [58, 151].

Other therapeutic strategies to target CAMP-induced inflammation have been extensively studied, as depicted in Fig. 2 and summarized in Table 3. For example, the neutralization of cfDNA through DNase and scavenger-molecule based approaches have been utilized. Previously, NABPs have been shown to scavenge proinflammatory nucleic acids and modulate inflammation at injured sites [153]. Specifically, third generation polyamidoamine dendrimer (PAMAM-G3) is a widely studied NABP that has demonstrated ability to prevent TLR activation in target immune cells through scavenging nucleic acid and nucleic-acid proteins in a multitude of acute inflammatory disease states, including liver failure, influenza infection, and cancer metastasis. To address the limitation of unwanted, in vivo cytotoxic effects of PAMAM-G3, a mesoporous silica nanoparticle functionalized with polyethylenimine (MSN-PEI), a nucleic acid-biding nanoparticle (NABN), was synthesized with the intent of improving toxicity profiles. Novel synthesized nanoparticles have similarly demonstrated the ability to scavenge cfDNA and ameliorate septic injury in experimental models of sepsis, including cecal ligation and puncture (CLP) [58]. Thus, further nanoparticulate NABNs-based scavenging approaches may provide a promising future therapeutic avenue for addressing cfDNA in lethal inflammatory disorders, including sepsis.

A different strategy that has been investigated is the use of membrane-coated cartridges to scavenge dead cells, pathogens, or specifically DAMPs from circulation. Early employment of this technique utilized polymyxin B immobilized to a polystyrene-derived fiber to remove circulating LPS. Using this scavenger cartridge, blood if filtered outside the patient using an extracorporeal circuit, thereby detoxifying blood and removing nearly 90% of circulating LPS. This therapeutic strategy has been implemented in septic patients with little reported adverse events, however further studies are needed to determine true clinical efficacy in improving outcomes [154]. Utilizing a similar strategy, certain types of NABPs, e.g., PANAM-G3, beta-cyclodextrin-containing polycation (CDP) and hexadimethrine bromide (HDMBr), immobilized onto an electrospun microfiber mesh were capable of capturing and removing extracellular DNAs as well as HMGB1 from circulation. NABP-immobilized mesh also neutralized the ability of DAMPs generated by ex vivo cell culture or DAMPs circulating in the blood of trauma patients to stimulate multiple TLRs in vitro and in vivo [155]. Thus, therapeutic approaches utilizing membranes coated with CAMP-capturing polymers may be a promising strategy during hemofiltration, extracorporeal membrane oxygenation (ECMO) and continuous renal replacement therapy (RRT) to scavenge CAMPs and ameliorate CAMP-induced inflammation in septic patients.

A broad variety of proteins can promote internalization of harmful molecules with subsequent pro- and anti-inflammatory impacts. Previously, it has been shown that HMGB1 can bind LPS and target macrophage internalization and delivery to lysosomes via the RAGE receptor. Although HMGB1 is permeabilized in the acidic environment of the lysosome, the impact of cytosolic LPS in this mechanism results in the activation of caspase-11, pyroptosis, and cell death in endotoxemia and bacterial sepsis [156]. DAMPs (such as HMGB1 and peroxiredoxins) are ligands for many other scavenger receptors that similarly promote internalization. For example, class A scavenger receptors (including MSR1) have been shown to facilitate macrophage internalization of HMGB1, but also that these receptors (MSR1 and MARCO) served as co-receptors for pro-inflammatory TLR4 signaling. In this same work, however, double scavenger receptor-(MSR1 and MARCO)-deficient mice still internalize HMGB1 efficiently, suggesting that other scavenger receptors or related molecules play a role in macrophage internalization [157]. On the other hand, it has been reported that clearance of DAMPs by class A scavenger receptors may provide anti-inflammatory impacts. For example, scavenger receptor-mediated clearance of DAMPs in a murine ischemic cerebral stroke model, largely mediated by MSR1, served to attenuate DAMP-mediated inflammatory signaling, thereby improving cerebral pathology [158]. Broadly, the role of class A scavenger receptors in inflammation is controversial, and likewise the resulting sequelae of this receptor-ligand interaction of scavenger receptors to DAMPs is not fully understood. Regardless, better understanding of these interactions and their relationship to the clearance of CAMPs in acute disease states may promote discovery of new therapeutic strategies in regulating CAMP-induced inflammation. Along with these scavenging mechanisms, a summary of methods for targeting CAMPs to attenuate inflammation and ALI in sepsis is shown in Fig. 3, and the preclinical evidence for each CAMP-specific therapeutic strategy is summarized in Table 3.

## Conclusions and future directions

Sepsis is a multifactorial inflammatory disease condition whose pathophysiology is enigmatic. Distinguishing CAMPs from the broad area of DAMPs may establish the notion that the source/origin of DAMPs matters for the differential intensities of inflammation in sepsis, further directing source control to regulate the release of CAMPs during sepsis. Given that various infectious insults can contribute to the progression of inflammation to sepsis, elucidating the unique roles of CAMPs in other modes of inflammation, including sterile inflammation, bacterial and viral-based inflammation, and disease-specific inflammation, are worthy areas of continued research. Furthermore, the number of molecules considered to be DAMPs is increasing, as a recent study unveiled a myriad of intracellular molecules released during LPS stimulation of macrophages through active and pyroptotic pathways. Our approach of grouping DAMPs into the unique category of CAMPs will further stimulate the creation of other new subcategories based on the characteristics or size of released molecules. Studies on the release and functions of CAMPs are mainly focused on immune cells in terms of inducing cytokine production and cellular heterogeneity. Future studies focused on the role of CAMPs on non-immune cells may also reveal new directions on cell-type specific effects of CAMP release and their mode of action in sepsis.

Several post-translational forms of HMGB1 have been identified, relying on the extracellular environmental pH among other factors. Modified extracellular HMGB1 (the redox state of cysteines 23, 45, and 106) exhibit different functions compared to their parent form [7]. Future studies on whether other CAMPs (like eCIRP and histones) show similar post-translational modifications induced by external environments will be of great value. During transcription, several transcriptional factors bind to regulatory elements. As ETs or cfDNA are released, there is a possibility that these transcriptional factors may also be released along with bound DNA. Identification of extracellular transaction factors and their functions on the immune system may uncover greater understanding in the disease pathophysiology of sepsis. Furthermore, during inflammatory responses, epigenetic changes of DNA and histones in cells are altered. Epigenetically modified CAMPs may exhibit differential outcomes in sepsis pathophysiology. Finally, these identified CAMPs may interact with one another (and other proteins) to form complexes once released extracellularly, in addition to the crosstalk between their downstream signaling pathways. Regarding the interplay of CAMPs, it has previously been shown that targeting one CAMP could abrogate inflammation and tissue injury by inhibiting pro-inflammatory mediators and other CAMPs [20]– highlighting the importance of the interrelationship among various CAMPs in sepsis. As CAMP interactions may confer great inflammatory consequences, studying these interactions may provide new targets in preventing dangerous inflammatory cascades in sepsis. Unveiling the unique category of these important endogenous molecules, CAMPs, in sepsis defines the pathophysiology of inflammatory disease and provides new therapeutic avenues in preventing and treating uncontrolled inflammation.

## Supplementary information

Reproducibility checklist

## Data Availability

There are no experimental datasets given that this is a review article that is prepared based on a literature review.
